# 4382 All IN for Health: Promoting good health and engaging a health research volunteer community in the Hoosier state

**DOI:** 10.1017/cts.2020.190

**Published:** 2020-07-29

**Authors:** Jessica Hall, Christine Drury, Carmel Egan

**Affiliations:** 1 Indiana University School of Medicine

## Abstract

OBJECTIVES/GOALS:

- To improve and expand health and research literacy throughout Indiana by sharing health-focused resources and research outcomes.
- To encourage and increase health research participation throughout Indiana by promoting health research opportunities, including clinical studies.

METHODS/STUDY POPULATION:

- Discover and understand community concerns and barriers to good health and clinical research participation by providing a platform for individuals and communities to share their voices.
- Educate Indiana residents on the importance of participating in health research.
- Engage with the community to meet them where they are (online) and continue to build relationships throughout the state.
- Promote healthy living for Indiana residents by sharing health education and resources from existing state health organizations and initiatives.
- Develop and maintain the largest statewide database of research volunteers.

RESULTS/ANTICIPATED RESULTS:

- The anticipated results from this program include engagement of all populations and all communities throughout the state in conversation and education around good health and health research, as well as participation in health research across the CTSI’s partner organizations. Large-scale growth is expected in both the online community and consented volunteer registry is expected to include and engage racially and ethnically diverse populations, as well as special health populations, such as representatives of rural communities, aged, rare disease survivors, and transgender individuals.

DISCUSSION/SIGNIFICANCE OF IMPACT:

- Thorough this work, the Indiana CTSI has developed a unique program, educating the public about health research and opportunities to participate, while simultaneously supporting research departments with marketing promotion of their efforts, and a ready statewide volunteer community.

